# Healthy Human T-Cell Responses to *Aspergillus fumigatus* Antigens

**DOI:** 10.1371/journal.pone.0009036

**Published:** 2010-02-17

**Authors:** Neelkamal Chaudhary, Janet F. Staab, Kieren A. Marr

**Affiliations:** 1 School of Medicine, Johns Hopkins University, Baltimore, Maryland, United States of America; 2 Sidney Kimmel Comprehensive Cancer Center, Johns Hopkins University, Baltimore, Maryland, United States of America; David Geffen School of Medicine at University of California Los Angeles, United States of America

## Abstract

**Background:**

*Aspergillus fumigatus* is associated with both invasive and allergic pulmonary diseases, in different hosts. The organism is inhaled as a spore, which, if not cleared from the airway, germinates into hyphal morphotypes that are responsible for tissue invasion and resultant inflammation. Hyphae secrete multiple products that function as antigens, evoking both a protective (T_H_1–T_H_17) and destructive allergic (T_H_2) immunity. How *Aspergillus* allergens (Asp f proteins) participate in the development of allergic sensitization is unknown.

**Methodology/Principal Findings:**

To determine whether Asp f proteins are strictly associated with T_H_2 responses, or represent soluble hyphal products recognized by healthy hosts, human T cell responses to crude and recombinant products were characterized by ELISPOT. While responses (number of spots producing IFN-γ, IL-4 or IL-17) to crude hyphal antigen preparations were weak, responses to recombinant Asp f proteins were higher. Recombinant allergens stimulated cells to produce IFN-γ more so than IL-4 or IL-17. Volunteers exhibited a diverse CD4+ and CD8+ T cell antigen recognition profile, with prominent CD4 T_H_1-responses to Asp f3 (a putative peroxismal membrane protein), Asp f9/16 (cell wall glucanase), Asp f11 (cyclophilin type peptidyl-prolyl isomerase) and Asp f22 (enolase). Strong IFN-γ responses were reproduced in most subjects tested over 6 month intervals.

**Conclusions:**

Products secreted after conidial germination into hyphae are differentially recognized by protective T cells in healthy, non-atopic individuals. Defining the specificity of the human T cell repertoire, and identifying factors that govern early responses may allow for development of novel diagnostics and therapeutics for both invasive and allergic *Aspergillus* diseases.

## Introduction

While our airways are exposed to the ubiquitous *Aspergillus fumigatus* (*Af*) constantly, only a subset of people develop complications; pulmonary manifestations fall into a spectrum of hypersensitivity respiratory disorders (Aspergillus induced asthma, allergic bronchopulmonary aspergillosis, allergic Aspergillus sinusitis, and hypersensitivity pneumonitis) to more invasive syndromes (pulmonary aspergillosis, disseminated disease). The immunopathogenesis of disease, and the nature of pulmonary host defenses- essentially, what defines the balance between destructive and protective inflammatory responses- are becoming better defined. The organism, inhaled as an inert, chitinous spore that is covered with hydrophobins, will germinate into germ tube and hyphal morphotypes if not cleared by resident phagocytic cells. Germination- and loss of the hydrophobin layer- results in exposure of β-glucan, a ligand for dectin-1, resulting in stimulation of inflammatory cytokines from macrophages and activation of specific maturation phenotypes on dendritic cells (DC) [1]–[5]. Hyphal morphotypes secrete multiple soluble products that are involved in microbial adaptation to the local environment (e.g. proteases, etc.). Effector phagocytes (neutrophils) are responsible for clearing hyphae; a subsequent T-lymphocyte response is triggered by DC traveling to lymph nodes for presentation of pathogen-produced antigens to naïve T cells. Thus, cells of innate immunity both function to clear the organism from the airways, and to initiate memory T cell responses. These CD4+ T lymphocyte responses can be both protective, with T-helper 1 (T_H_1) and T_H_17 phenotypes, and pathologic, with a predominant T_H_2 phenotype driving allergic inflammation.

What dictates generation of T_H_1–T_H_17 or T_H_2 immunity is becoming more understood, with results of recent studies outlining the pivotal role of microbial adjuvants in tailoring the activating phenotypes on antigen presenting cells. DC stimulated through dectin-1 activate CD4+ T_H_1 and T_H_17 cells [5]; similarly, DC that are stimulated via dectin-1 efficiently prime cytotoxic T cells [6]. Other microbial adjuvants, such as chitin, alternatively activate macrophages to drive T_H_2 immunity [7]. The nature of the protein antigen recognized likely also serve to promote a specific inflammatory phenotype; recent studies have shown that basophils serve as antigen presenting cells to augment T_H_2 immunity, particularly in response to soluble proteases [8], [9].

*A. fumigatus* appears particularly adept at promoting T_H_2-immunity and allergic inflammation, and several secreted ‘allergens’ have been described. These proteins, also called “Asp f” proteins, were largely identified as proteins recognized by CD4+ T_H_2 cells or IgE in allergic individuals. Homology-based sequence analyses indicate that these gene products include proteases, peptidases, and glucanases. It has been assumed that these proteins naturally evoke T_H_2 immune responses, with specific “allergenic” potential (thus the “Asp f” designations). However, these products also preferentially represent those that are abundantly expressed by the hyphal form of the organism, likely reflecting their activity in stress response and adaptation to the local environment [10]. We hypothesized that the Asp f proteins are not strictly functioning as allergens, but are the soluble *Aspergillus* products that are most abundantly recognized in the host after exposure to metabolically active hyphae. Answering this question is important both to our understanding of the immunopathogenesis of allergic diseases and to the development of assays to enable measurement of organism specific immune reconstitution. To address this, T cell responses to crude *A. fumigatus* hyphal antigens, and specific recombinant Asp f antigens were characterized in a cohort of healthy human volunteers. Results demonstrate that peripheral blood from healthy human volunteers contain CD4+ and CD8+ T cells with specificity to multiple hyphal-secreted antigens previously identified as allergens. These findings are consistent with the current paradigm in which efficacy of airway conidial clearance, and the nature of local inflammatory responses tailor induction and maintenance of specific immunity, not exposure to specific Aspergillus allergens.

## Results

### Expression of Recombinant Asp f Proteins

Multiple proteins have been described as “Asp f” allergens; these entries were tabulated from www.allergen.org, with homology based sequence comparison to more fully characterize the open reading frame and assign putative functions. In this analysis, we found that the allergens previously described as Asp f9 and Asp f16 corresponded to the same open reading frame (ORF). Multiple attempts to clone the cDNA for allergen Asp f16 were made, without success. Sequence information at GenBank (accession number AF062651) was used to generate oligonucleotides to amplify by PCR the entire cDNA for Asp f16 from the *A. fumigatus* sequenced strain, Af293 [11] and from the strain used by Banerjee, et al. (AF-102, ATCC 42202) in the original Asp f16 cloning [12]. Four and five independent clones from Af293 and ATCC 42202, respectively, were sequenced and found to be identical to the Asp f9 sequence (GenBank accession number AJ223327; this is a partial clone), now annotated as the cell wall glucanase Crf1 by TIGR (http://www.tigr.org/tdb/e2k1/afu1/afu1.shtml; genome locus AFUA_1G16190). Oligonucleotides designed with Asp f9 sequence information from TIGR only yielded Asp f9 cDNA PCR amplicons from ATCC 42202 cDNA. Hence, the product Asp f 9 is the same as Asp f 16, as designated here as Asp f9/16.

Either partial, or complete ORFs for cloning into the *E. coli* expression system were amplified (Table 1). Out of 17 proteins, eight were not expressed and one, Asp f1, a potent ribotoxin, could not be successfully cloned downstream of the *trp-lac* promoter, although the plasmid vector harbors the *lacI^q^* repressor gene to maintain regulation of the promoter in the absence of inducer. Upon closer examination of the deduced function of Asp f4, 5, 7, 8, 10, 13, and 18, four have been ascribed as proteases and the remaining proteins have unknown function (Table 1, gray shaded proteins). Multiple attempts to express these recombinant proteins were not successful, suggesting that these proteins were deleterious and or toxic to *E. coli* using the pTrcHis2 expression system. High level expression of Asp f2 was not achieved in this system even after induction in BL21(DE3) *E. coli* (Invitrogen) to minimize proteolysis of the recombinant protein (data not shown), and other attempts to increase production to levels amenable for bioassays were unsuccessful.

**Table 1:** Asp f proteins.

Allergen	GenBank entry	Calculated size	Genomic locus	Putative function
Asp f1*	M83781	19.6 kDa	Afu5g02330	major allergen I; ribotoxin
**Asp f2**	**U56938**	**32.8 kDa**	**Afu4g09580**	**allergen 2**
**Asp f3**	**U58050**	**18.4 kDa**	**Afu6g02280**	**peroxisomal membrane protein**
Asp f4	AJ00173	30.0 kDa (**34.0 kDa**)#	Afu2g03830	allergen 4
Asp f5	Z30424	68.7 kDa	Afu8g07080	metalloprotease (MEP)
**Asp f6**	**U53561**	**23.4 kDa**	**Afu1g14550**	**manganese superoxide dismutase**
Asp f7	AJ223315	11.6 kDa (**27.5 kDa**)	Afu4g06670	allergen 7
Asp f8	AJ224333	11.1 kDa	Afu2g10100	acidic ribosomal protein P2
**Asp f9/16**	**AJ223327**	**32.3 kDa (40.2 kDa)**	**Afu1g16190**	**cell wall glucanase Crf1**
Asp f10	X85092	41.6 kDa	Afu5g13300	aspartic endopeptidase Pep1
**Asp f11**	**AJ006689**	**19.5 kDa**	**Afu2g03720**	**cyclophilin type peptidyl-prolyl isomerase**
**Asp f12**	**U92465**	**50.5 kDa (80.3 kDa)**	**Afu5g04170**	**Heat shock protein hsp90 family**
Asp f13	AJ002026	15.9 kDa	Afu2g12630	allergen 13/cerato-platanin (protease)
**Asp f17**	**AJ224865**	**19.4 kDa (27.3 kDa)**	**Afu4g03240**	**galactomannoprotein MP1/B-glucanase**
Asp f18	Y13338	52.6 kDa	Afu5g09210	alkaline serine protease Alp2
Asp f22	AF284645	47.3 kDa	Afu6g06770	enolase
Asp f23	Q8NKF4	44.4 kDa	Afu2g11850	60S ribosomal protein L3

* Asp f1 cDNA could not be cloned downstream of the pTrc promoter in pTrcHis2.

#Molecular sizes in bold correspond to the full length protein.

Proteins in bold were successfully expressed in *E. coli*.

**Figure 1:**
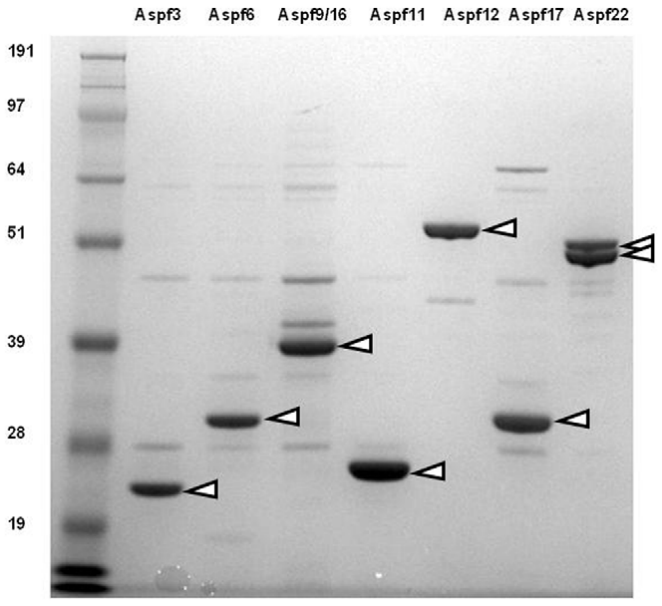
Recombinant Asp f proteins expressed in *E. coli*. SDS-PAGE of Asp f3, Asp f6, Asp f9/16, Asp f11, Asp f12, Asp f17, and Asp f22 produced in *E. coli* with C-terminal *c-myc* and His_6_ tags. Each lane contains one microgram of protein. Triangles identify Asp f proteins. Asp f22 appears as a doublet due to loss of the C-terminal tags in the faster migrating protein species. Molecular size standards (SeeBlue Plus2 Pre-Stained Standards, Invitrogen) are shown at left in kD.

Recombinant Asp f12 was prone to aggregation and precipitation, and subsequently purified by Ni^2+^ chelate chromatography as per the other recombinant Asp f proteins (Materials and Methods), but under denaturing conditions in the presence of 6 M guanidine HCl (Sigma Aldrich) according to established protocols [13], [14]. The remaining Asp f proteins (Asp f3, Asp f6, Asp f9, Asp f11, Asp f17, and Asp f22) were readily expressed at high levels in *E. coli* (Figure 1); these 7 proteins were used in subsequent assays to measure T cell responses.

### Healthy Humans' Response to Crude and Recombinant *Af* Antigens

**Figure 2:**
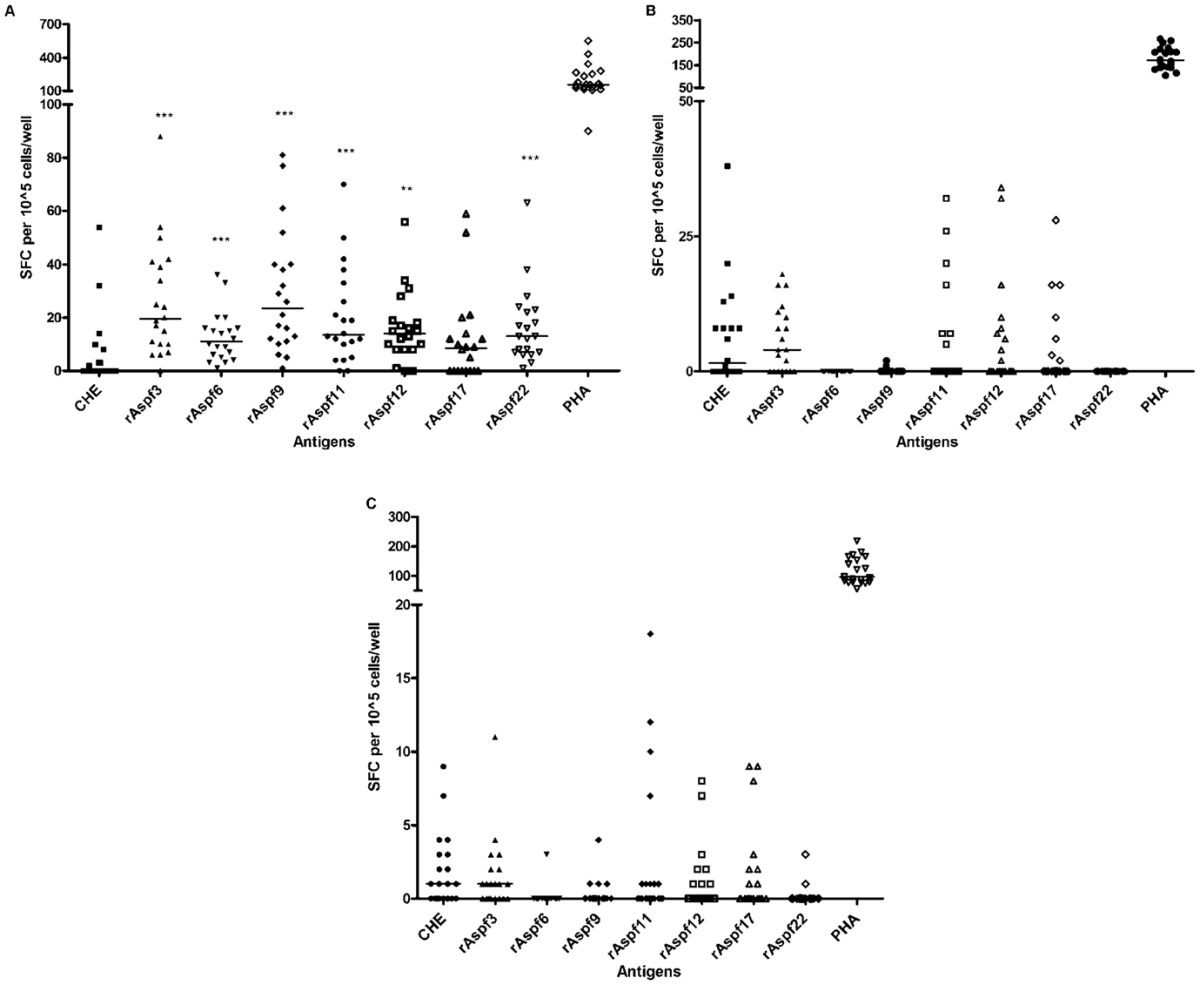
ELISPOT assays using *A. fumigatus* crude hyphal extract (CHE) and purified recombinant antigens. (a) IFN-γ (b) IL-4 and (c) IL-17 ELISPOT assay. Blood was collected from 20 healthy donors and triplicate samples of 1×10^5^ (IFN-γ) or 2×10^5^ PBMCs (IL-4 or IL-17) were stimulated with medium, 5 µg/ml phytohemoagglutinin (PHA) or 1 µg/ml CHE or recombinant *A. fumigatus* antigens for 24–48 hrs at 37°C/5%CO_2_. The mean antigen-specific spot forming cells, SFC (after background subtraction of control wells with no antigen) is plotted. The median for each set of healthy individuals is indicated by a bar. The figures show one representative data from three independent experiments. ^***^ *P*<0.001, ^**^ *P*<0.01 versus CHE stimulated PBMC.

PBMC from 20 healthy individuals were tested against a panel of seven *E.coli*-expressed recombinant *Af* antigens (Asp f3, Asp f6, Asp f9, Asp f11, Asp f12, Asp f17 and Asp f22) and crude hyphal extract (CHE) using ELISPOT. Healthy donors' PBMC exhibited variability of IFN-γ and IL-4 responses, as shown by robust or weak responses to *A. fumigatus* antigens (i.e. SFU varying from zero to 80, Figure 2a, b). Few IL-17 producing cells were detected using this assay, from any donors (Figure 2c).

All recombinant proteins elicited some measurable IFN-γ producing cells, although the range of SFU varied widely (Figure 2a). rAsp f3, rAsp f11, rAsp f12, and rAsp f17 elicited detectable IL-4 producing cells, although the numbers were low in most donors (Figure 2b). Crude hyphal extract (CHE) induced weak IFN-γ, IL-4, and IL-17 responses when compared to all recombinant proteins (Figure 2a–c).

Table 2 demonstrates the relative quality of responses (strong, weak, and none) among 20 volunteers. Few donors exhibited strong IFN-γ responses to CHE; in contrast, rAsp f3, rAsp f9/16, rAsp f11 and rAsp f22 elicited relatively higher IFN-γ production. These antigens did not induce IL-4 producing cells from the same donors; few recombinant antigens were associated with the strong IL-4 responses. None of the volunteers had strong IL-17 responses to the antigens tested, although some people had small numbers of measurable IL-17 circulating cells, particularly in response to CHE and rAsp f3 (Table 2).

**Table 2:** IFN-γ, IL-4 and IL-17 T-cell responses of healthy donors (n = 20) to CHE and recombinant antigens of *A. fumigatus*.

Antigens	IFN-γ			IL-4			IL-17		
	SR	WR	NR	SR	WR	NR	SR	WR	NR
**CHE**	2	5	13	2	9	9	0	12	8
**rAsp f3**	10	9	1	0	14	6	0	12	8
**rAsp f6**	4	16	0	0	0	20	0	1	19
**rAsp f9/16**	11	9	0	0	2	18	0	4	16
**rAsp f11**	7	11	2	3	4	13	0	9	11
**rAsp f12**	4	14	2	2	7	11	0	8	12
**rAsp f17**	4	8	8	1	6	13	0	8	12
**rAsp f22**	6	14	0	0	0	20	0	2	18

Strong responders (SR) were defined ≥20 SFU/10^5^ cells, weak responders <20 SFU/10^5^ cells. NR  =  no SFU quantified. Data are derived from means of triplicate measurements in three independent experiments.

**Figure 3:**
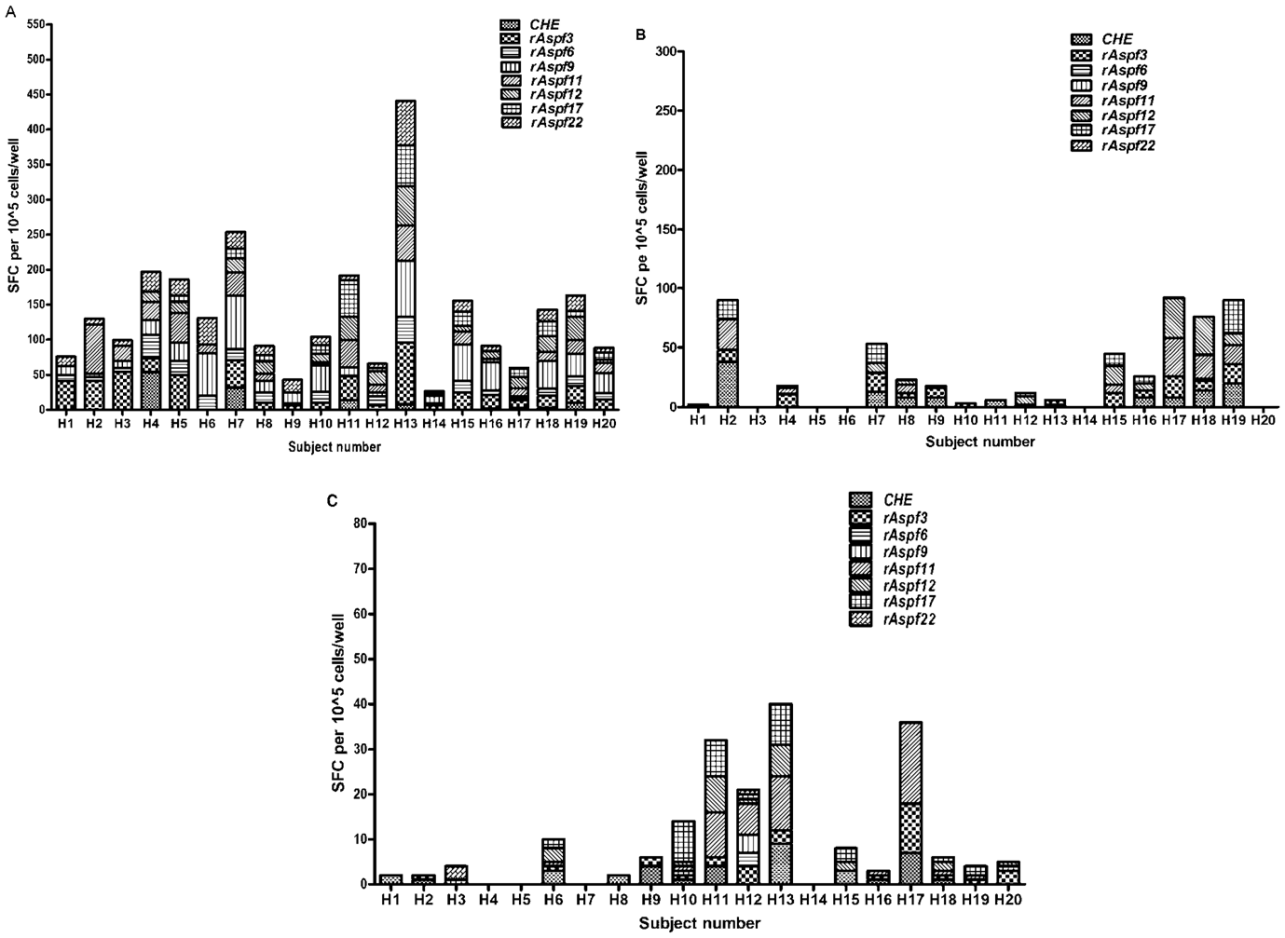
*A. fumigatus* antigens recognition by 20 healthy donors. (a) IFN-γ (b) IL-4 and (c) IL-17 responses. Blood was collected from 20 healthy donors and triplicate samples of 1×10^5^ PBMCs were stimulated with medium, 5 µg/ml PHA or 1 µg/ml CHE or recombinant *A. fumigatus* antigens for 24–48 hr at 37°C/5%CO_2_. The mean antigen-specific spot forming cells, SFC (after background subtraction of control wells with no antigen) is plotted.

The repertoire of antigen recognition was characterized in 20 volunteers (Figure 3). Four of 20 donors (H4, H7, H11 and H13) had strong IFN-γ responses to all of the recombinant *Af* antigens (Figure 3a). In donor H7, IL-4 secreting T-cells were also strong after challenges with all rAsp f proteins, however, measured responses were weaker when compared to IFN-γ (Figure 3a, b). CHE stimulated IFN-γ producing cells strongly in donors H4 and H7; two subjects (H2 and H19) had strong CHE-induced IL-4 responses (Figure 3a, b). Weaker IL-17 responses (ELISPOTs <20, varying from 10–18 spots) were observed with rAsp f11 in donors H11, H12, H13 and H17 (Figure 3c). Overall, these data suggest that the repertoire of T cells that recognize *Aspergillus* antigens is diverse, with variable antigen specificity; no obvious immunodominant antigens were identified, although certain recombinant Asp f proteins were more frequently associated with strong IFN-γ responses.

**Figure 4:**
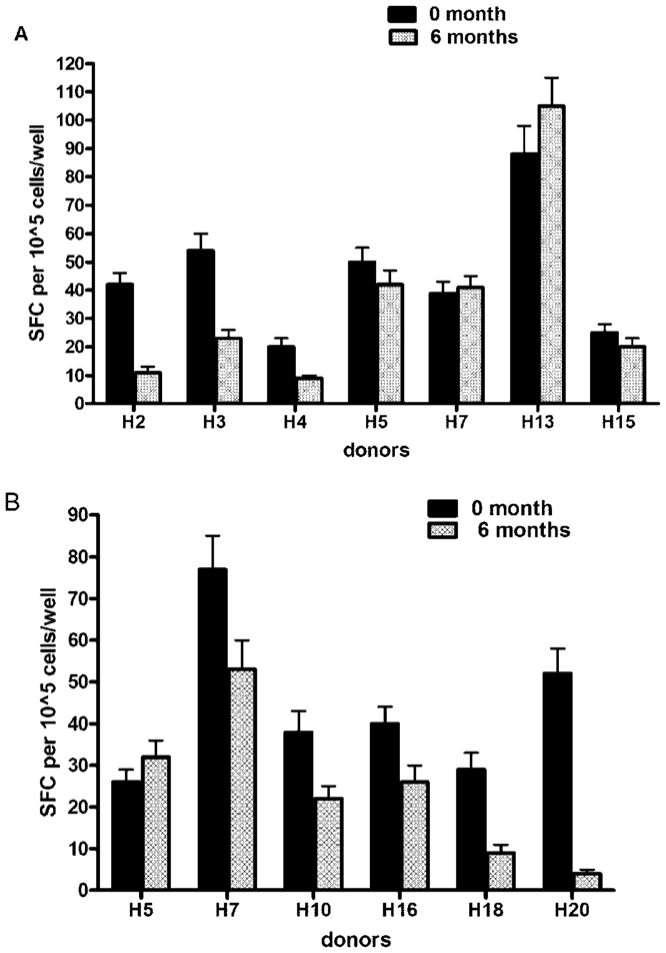
IFN-γ stability assay with rAsp f3 and rAsp f9 after period of 6 months. To determine whether the IFN-γ responses are stable over time, blood was collected from high responders after six month interval of previous assay and triplicate samples of 1×10^5^ IFN-γ PBMC were stimulated with medium and 1 µg/ml rAsp f3 (a) or rAsp f9 (b) for 18–24 hrs at 37°C/5%CO_2_. The mean antigen-specific spot forming cells, SFC (after background subtraction of control wells with no antigen) ± SD is shown. The figures show one representative data from three independent experiments.

Stability of IFN-γ responses were examined in a subset of strong responders, using PBMC collected at different time points, separated by 6 months. The two strongest antigens stimulating IFN-γ producing cells, rAsp f3 and rAsp f9/16, were tested (Figure 4a). Strong responses were maintained in 5 of 7 donors (H3, H5, H7, H13, and H15) in response to rAsp f3. Four of six donors (H5, H7, H10, and H16) also had stably strong responses after rAsp f9/16 challenge (Figure 4b).

### Recombinant Asp f3 and Asp f9 Are Recognized by Both CD4+ and CD8+ T-Cells

**Figure 5:**
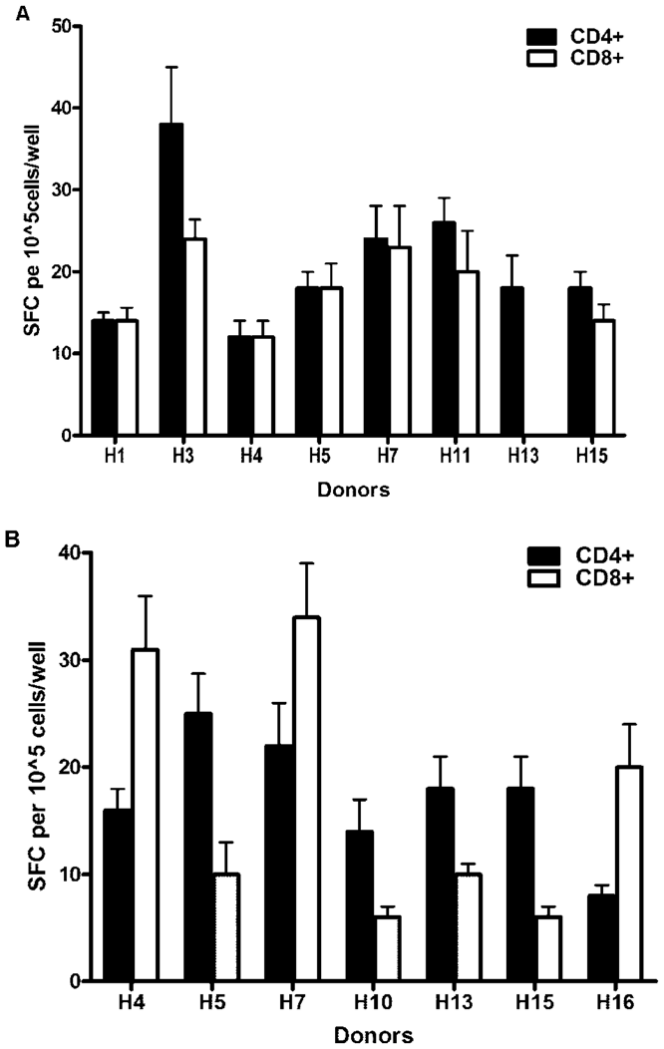
IFN-γ ELISPOT assay with purified CD4^+^ and CD8^+^ T-cells. CD4^+^ or CD8^+^ T-cells (1×10^5^) were isolated from strong responders' PBMC, incubated with 1 µg/ml of (a) rAsp f3 and (b) rAsp f9 with autologous irradiated PBMC (5×10^4^) in triplicates for 24 hr at 37°C/5% CO_2_. PHA (5 µg/ml) was used as a positive control. The mean antigen-specific spot forming cells, SFC (after background subtraction of control wells with no antigen) ± SD is shown.

Negative selection was performed to determine whether IFN-γ producing cells comprising responses to rAsp f3 and rAsp f9/16 were CD4+ or CD8+. Donors who exhibited the strongest responses were tested (Figure 5). Only one of eight strong responders tested to rAsp f3 had single lineage CD4+ responses (H13), and all seven tested to rAsp f9/16 had mixed CD4+ and CD8+ T cells (Figure 5).

## Discussion

CD4+ T lymphocytes are important mediators of host inflammatory/anti-inflammatory responses, with the balance between T_H_1/T_H_17 and T_H_2 phenotypes dictating disease pathology. *Aspergillus fumigatus* enters the airway as conidia, which are not inflammatory, until loss of a hydrophic coat [1]–[4]. In the presence of inadequate clearance, organisms germinate into hyphae, which expose multiple cell-associated and soluble products that are recognized to promote inflammation and to serve as antigens/allergens in the resultant early- and late-phase inflammatory reactions [15]. Chronic inflammatory diseases result in response to certain inhaled allergens, which are processed to T cells that exhibit a predominant T_H_2 phenotype, leading to Ig subtype switching and IgE synthesis, mast cell degranulation, eosinophilia, mucus metaplasia and airway hyperresponsiveness. Multiple microbial factors promote development of T_H_2-inflammation, including the influence of certain microbial adjuvants during antigen presenting cell activation, and the nature of the protein itself. *Aspergillus* ‘allergens’ have been detected as proteins recognized in patients who have hypersensitivity syndromes; results of this study suggest that at least some of these proteins are not strictly allergens, but induce T_H_1 cytokine responses in non-atopic individuals, thus emphasizing the overlapping capacity of hyphal proteins to activate both protective and non-protective inflammation.

Most *A. fumigatus* proteins that serve as allergens have been identified from screens identifying proteins that bind IgE antibody or, less commonly, stimulate CD4+T_H_2 clones in people with asthma or ABPA [16]. These allergens have been classified as having multiple diverse putative functions, including activity in hydrolysis and breakdown of local tissues, transport, and regulatory heat-shock proteins [16]. Structure-function properties of *Aspergillus* allergens have not been defined; it is possible that many of these proteins are distinguished as allergens by virtue of the abundance of in vivo expression, and structural presence of B-cell and T-cell epitopes. Results of this study show that healthy, non-atopic people also have a variable, and diverse profile of circulating T_H_1-producing CD4+ and CD8+ T cells that recognize Asp f products. Hence, the human T cell repertoire to this fungus appears diverse, but with a predilection towards responses developed to hyphal and soluble products. These findings are consistent with the current paradigm in which that nature of CD4+ responses (T_H_1–T_H_17 vs. T_H_2) are largely dictated by the inflammatory milieu, not singularly the function of the expressed antigen per se.

Proteomic data suggests that some Asp f proteins are highly expressed by mycelia in vitro [10]. Three of the 7 Asp f products that elicited strong T_H_1 type responses in healthy subjects screened here (Asp f3, Asp f12, and Asp f22) are some of the most highly abundant proteins expressed by mycelia. Hence, it is likely that at some point in life, these healthy volunteers were exposed to hyphal products, yet did not develop overt pulmonary invasive or allergic disease.

Interest in establishing assays to measure Aspergillus-specific immune reconstitution has been increased in recent years, with recent studies demonstrating the importance of T_H_1-type CD4+ T cells in mediating protection and recovery from invasive pulmonary aspergillosis [17]–[19]. However, identification of immunodominant antigens to enable accurate measurement of protective responses- and to potentially develop immunotherapies- has been elusive. Many studies have used crude hyphal extracts or antigen formulations to measure PBMC responses or to generate antigen-specific cell lines [20], [21]. Results of studies shown here suggest that these formulations may be suboptimal in measurement of PBMC responses. This is consistent with results from Stanzani *et al.*, who reported that healthy donors demonstrate weak functional T-cell responses to *A. fumigatus* crude extracts [22]. Hyphal extracts contain gliotoxin, an immunosuppressive mycotoxin, which induces apoptosis of antigen-presenting cells and inhibits transcription factor NF-κB [22]–[24] As these formulations contain multiple microbial adjuvants (β-glucan, chitin) and inhibitory molecules, standardization of antigen preparations is necessary to allow for reproducibility and transparency in study results.

While we observed that healthy volunteers have circulating CD4+ and CD8+ T cells reactive to multiple recombinant proteins, we do not know which proteins, if any, are ‘protective’ or dominant in the human T cell repertoire. However, studies in animal models and humans suggest that the two gene products eliciting most robust strong responses in healthy volunteers (Asp f3 an Asp f9/16) are candidates of interest for vaccine development. Naïve immunocompetent mice immunized with viable *A. fumigatus* conidia had IgG antibodies predominantly to Asp f3 antigen [25]. The Asp f3 gene (a putative peroxismal membrane protein) is homologous to the protective and dominant *Coccidioides posadasii* antigen pmp1 [26]. Also, in this study, recombinant antigen Asp f9 (mistakenly identified as Asp f16 in earlier studies) induced IFN-γ responses with weaker recognition by IL-4 or IL-17-secreting T-cells. Using murine models of invasive aspergillosis, Bozza *et al* have shown that intranasal delivery of Asp f9 (reported as Asp f16) with unmethylated CpG oligodeoxynucleotides induces protective T_H_1 response against *A. fumigatus* [27]. Dendritic cells pulsed with overlapping pentadecapeptides from Asp f9 (reported as Asp f16) induced IFN-γ secretion by both HLA-class I and class II-restricted T-cells in humans [28], [29]. Finally, a recent study by Bozza and colleagues found that Asp f9 gene product (Crf1p, Afu1g16190) appeared to retain an immunodominant T_H_1/Treg activating potential in both mice and healthy humans [4].

There are likely to be multiple other secreted and membrane-associated molecules that are sensed by human CD4+ T cells during development of a protective response. Bozza and colleagues described Cat1p and Pep1p (Asp f10) and the anchored protein Gel1p as highly recognized by human IFN-γ CD4+ T cells [4]. We also observed that the *A. fumigatus* enolase, rAsp f22, was recognized exclusively by IFN-γ secreting T-cells. Asp f22 is known to have a homologue in *C. albicans* that has been reported to induce protective response against systemic candidiasis in mice [30], [31]. More studies are necessary to fully characterize the human T cell repertoire in healthy and allergic people.

The cohort contained strong responders to rAsp f3 and rAsp f9, with both CD4+ and CD8+ T cell responses suggesting both HLA class I and class-II restricted epitopes in these antigens. DCs cross-present fungal antigens to stimulate protective and damaging CD8 T cell responses to multiple fungal pathogens, including *Pneumocystis* species, *Cryptococcus* species, and *Histoplasma capsulatum*, among others [11], [32], [33]. CD8+ T cells are recruited to the lungs in the context of *A. fumigatus*-induced inflammation [34], although the function of cytotoxic T cells in anti-Aspergillus immunity and mediating the inflammatory/anti-inflammatory balance is not known. HLA-class I and II-restricted overlapping peptides from Asp f9 (reported as Asp f16) induced IFN-γ producing CD4^+^ and CD8^+^ T-cells, respectively with cytotoxicity to *Af* culture filtrate-pulsed target cells have been reported [28], [29].

It is assumed that most healthy donors have roughly equivalent exposure to hyphal antigens, in a dynamic daily process of conidial airway clearance. However, we measured great variability in the quantity, and quality of the T-cell responses to *A. fumigatus* recombinant antigens. This observation is consistent with results of multiple studies implicating genetic haplotypes and/or polymorphisms in mediating innate and acquired immunity. For instance, responses may be attributed to the specific HLA-haplotypes or polymorphisms in other genes whose products influence the inflammatory nature of early innate immune responses (e.g. TNF-α, TLR4) [35]–[37].

The quantity of measured *Aspergillus*-specific ELISPOT responses in this healthy cohort are not robust when compared to select viral antigens such as cytomegalovirus, in which CMV-seropositive people develop higher quantities of measurable IFN-γ producing cells, in a dynamic interplay between intermittent viral reactivation and augmentation of immunity [38], [39]. However, the bulk of donors tested did show relative stability of “strong” responses when tested over time. While variation of responses may be caused by time-dependent changes in antigen exposure, this would be difficult to measure. More studies need to be performed to determine the relative kinetics of responses, and implications in the context of secondary immunosuppression.

There are limitations to the conclusions that can be drawn in this study. Only a few allergens were tested, in a relatively small cohort of healthy volunteers. Also, we were unable to express some products in the *E. coli* system, despite multiple attempts to minimize potential cytotoxicity and proteolytic activity. Many of the products that were not expressed are putative proteases, which would have been of particular interest given functional associations between protease activity and T_H_2 immunity [40]. High level expression of some products has been achieved in a Baculovirus system [41], in *E. coli* using a pET 23b (+) vector [27], [42], and in *S. cerevisiae* [4]. Further studies are warranted as lack of glycosylation may alter the quantity or nature of responses measured. While preparations were confirmed to contain <0.1 EU/mL endotoxin, there exists a possibility that low (not measurable) amounts of LPS could function to induce nonspecific responses. We believe that this is unlikely as *E. coli* protein controls did not elicit responses.

In summary, results of this study demonstrate that some *Aspergillus* allergens function as antigens to T_H_1-directed CD4+ and cytotoxic T cells, with healthy volunteers demonstrating a diverse repertoire to multiple hyphal expressed proteins. This supports the paradigm in which local inflammatory responses, and the nature of chronic immunity, is tailored by multiple microbial and host variables dictating functional phenotypes. Identification of dominant and protective antigens, and development of robust assays for performance of immune reconstitution studies awaits further studies.

## Materials and Methods

### Peripheral Blood Mononuclear Cell Isolation of Healthy Humans

Healthy donor blood samples were obtained under protocols approved by the Fred Hutchinson Cancer Research Center, Oregon Health and Sciences University, and Johns Hopkins University Institutional Review Boards following written informed consent. Peripheral blood was drawn from 20 healthy donors (Men = 11, Women = 9), between the ages of 27 to 46 years. No volunteers had a history of suspected or proven fungal infection or reported allergy/atopy. Peripheral blood mononuclear cells (PBMC) were separated by use of Ficoll-Hypaque density gradient centrifugation (Histopaque-1077, Sigma, St Louis, MO), washed twice in phosphate-buffered saline (PBS) (GIBCO Invitrogen Corp.), and resuspended with RPMI-1640 medium supplemented with 10% Fetal Bovine serum (GIBCO Invitrogen Corp.) and penicillin-streptomycin (GIBCO Invitrogen Corp.). All cell preparations were more than 95% viable as judged by trypan blue exclusion.

Isolated PBMC were suspended in cold freezing medium (10%DMSO/90%FBS) at a density of 5–6 million cells per ml, and aliquots were frozen at −80°C for 24 hrs before transfer to liquid nitrogen. To revive PBMC, cryovials were immediately thawed in a 37°C water-bath, washed twice in 10% RPMI-media and viability was assessed by trypan-blue exclusion. This method yielded recovery of 90–95% cells from frozen PBMC.

### Preparation of *Aspergillus fumigatus* Crude Hyphal Extract (CHE)

*A. fumigatus* crude hyphal extract (CHE) was generated by culture of Af293 isolate (sequenced strain provided by D. Denning, University of Manchester, UK) (5 days, 37°C) in RPMI 1640 (+10% FCS). The hyphal mat was harvested, sequentially washed with PBS, and lysed by mechanical disruption with glass beads in a BeadBeater homogenizer (BioSpec Products, OK). The slurry was subjected to paraformaldehyde (1%) for microbial inactivation. Protein concentration was measured using the Bradford assay (Coomassie Protein Assay, Pierce) according to manufacturer's instructions. The product was concentrated using an endotoxin-free dialysis membrane with 5-kDa pore size (Pierce) to achieve a final protein concentration of 800–1000 µg/ml.

### Expression of Recombinant *A. fumigatus* Allergens in *E. coli*

Candidate *A. fumigatus* proteins were identified at the www.allergen.org database (Table 1). The sequence data for each allergen was verified against the Af293 *A. fumigatus* genome (www.sanger.ac.uk/Projects/A_fumigatus/) by BLAST analysis to ensure that these proteins were not strain-specific, as many of these antigens were initially identified and characterized as allergens of strain ATCC 42202 [43].

PCR amplification and cloning of cDNAs proceeded as follows: PCR oligonucleotides pairs were designed to amplify the entire (or partial) cDNAs without start and stop codons to optimize expression from pTrcHis2 (Invitrogen), and to fuse the C-termini to a *c-myc* epitope and 6X-His tags within the vector. Total RNA was prepared from Af293 grown in glucose minimal medium (MMG) [44] for 48 hr at 37°C, using the TRIzol reagent (Invitrogen), as per the manufacturer's recommendations with a slight modification (J.W. Bok and N.P. Keller, University of Wisconsin, personal communication). After TRIzol phase separation, the aqueous phase was extracted once with equal volume of phenol/chloroform/isoamyl alcohol (25∶24∶1), before RNA precipitation with isopropanol. Three milligrams of total RNA was used to isolate mRNA by binding to oligo(dT) cellulose as per the manufacturer's recommendations (FastTrack 2.0, Invitrogen). Four micrograms of poly A^+^ RNA was subsequently used in a cDNA synthesis reaction (Universal Riboclone System, Promega) primed with oligo(dT) to generate double-stranded cDNA templates. Each 50 µL PCR contained oligonucleotide pairs at 0.2 mM each, dNTPs at 0.2 mM each, MgCl_2_ at 1.5 mM, approximately 1.0 ng of cDNA, 1.0 unit of Platinum *Taq* DNA polymerase, in 1X PCR buffer (Invitrogen). The amplicons were generated by incubation in a thermal cycler for 2 min. at 94C, followed by 35 cycles of 94C for 1 min., 55C for 1 min. and 72C for 3 min. The reactions were terminated by a final extension of 72C for 12 min. and holding at 4C. Generation of amplicons of the expected molecular sizes was verified by gel electrophoresis and ethidium bromide staining as per usual methods [45]. The resulting amplicons were subsequently cloned into pTrcHis2 TOPO, and propagated in *E. coli* TOP10 cells (Invitrogen). The authenticity of the recombinant plasmids was verified by sequencing the cloned DNA across the junction sites between the insert and the vector.

Expression of His_6_-tagged proteins was induced in TOP10 *E. coli* grown to an OD_600_ of 0.6 in Luria broth [45] at 37C with 1 mM IPTG for 4 hr. Post induction, the cells were collected by centrifugation and stored frozen at −80C. Cell extracts were prepared by suspending the cell pellets in 3.0 mL of B-Per II Bacterial Protein Extraction Reagent (Pierce) with 1X Halt-EDTA free protease inhibitor cocktail (Pierce), and incubating at room temperature with rocking for 20 min. The cell debris was removed by centrifugation (27k×g for 15 min. at 4C), and the clarified extract was applied (100 µL) to three rows of a 96-well plate harboring SwellGel Ni^2+^ chelated discs (Pierce). The flow through was reapplied to the nickel-chelate slurry and the second flow through discarded. Remaining unbound proteins were removed by washing the nickel resin 4X with 200 µL of 50 mM Tris-Cl pH 8.0, 300 mM NaCl, and 50 mM imidazole. Bound proteins were eluted by adding 100 µL of Tris-Cl pH 8.0, 300 mM NaCl, 250 mM imidazole and collecting the flow through. The elution was repeated with new elution buffer, and the eluates pooled. The samples were subsequently desalted by exclusion chromatography over 5 mL Zebra Desalt spin columns, as recommended by the manufacturer (Pierce). Recovery and purity of the recombinant proteins were analyzed by SDS-PAGE (NuPAGE 10% or 12% acrylamide gels, Invitrogen) and staining (Bio-Safe Coomassie reagent, BioRad Laboratories, CA). Expression of the desired proteins was confirmed by their predicted molecular sizes in SDS-PAGs. Lastly, the samples were concentrated in Amicon centrifugal filter devices (Millipore), and quantified by the Bradford assay (Coomassie Protein Assay, Pierce) with BSA as standard. The concentrated recombinant protein preparations were deemed ≥80% pure by densitometric analysis of Asp f protein bands in relation to contaminating *E. coli* proteins in SDS-PAGE (data not shown). Gel electrophoresis images were captured with a GelDoc-ItTS Imaging System (UVP, Upland, CA), and manipulated with ImageJ processing and analysis software (http://rsb.info.nih.gov/ij/). Proteins preparations were confirmed to contain <0.1 EU/mL using the *Limulus* amebocyte lysate (LAL) reaction kit (E-Toxate; Sigma, St Louis, MO).

### ELISPOT Assays

ELISPOT assays were performed as previously described [46]. Briefly, ELISPOT plates (Multiscreen-MAIPS4510, Millipore) were coated using primary monoclonal antibodies against IFN-γ (Clone 1-D1K, MabTech, Sweden), IL-4 (82.4, MabTech, Sweden) and IL-17 (clone eBio64CAP17, eBioscience, San Diego, CA) at the concentration of 10 µg/ml in bicarbonate coating buffer, pH-9.6. PBMC (1×10^5^ cells/well for IFN-γ and 2×10^5^ cells/well for IL-4 and IL-17) in 10% RPMI media were added to the wells. Crude hyphal extract (CHE) and recombinant *A. fumigatus* antigens (Asp f3, Asp f6, Asp f9, Asp f11, Asp f12, Asp f17, and Asp f22) were added at the final concentration of 1 µg/ml, while cells alone were considered as negative control. Phytohemoagglutinin (PHA) was added at the concentration of 5 µg/ml as a positive control. IFN- γ ELISPOT plates were incubated at 37°C/5%CO_2_ for 24 hr, and IL-4 and IL-17 plates were incubated for 48 hr. Secondary antibodies to IFN- γ, IL-4 and IL-17 were added, and ELISPOT plates were developed using BCIP/NBT solution (MabTech, Sweden). Plates were dried and spots were enumerated using a Bioreader 4000 (Pro-X, BIOSYS GmbH, Germany). Results are shown as mean antigen-specific spot forming cells (SFC) after background subtraction of control wells containing no antigen.

To further characterize T cell responses to certain antigens, CD4+ and CD8+ T cells were purified from PBMC by negative selection using magnetic beads, according to manufacturer's instructions (Miltenyi Biotech, Auburn, CA). Purity was more than 90% as assessed by flow cytometry. Responder CD4^+^ or CD8^+^ T-cells (1×10^5^) were incubated with autologous irradiated PBMC (5×10^4^) in triplicates with PHA (5 µg/ml) or recombinant antigens (1 µg/ml) for 24 hr at 37°C/5% CO_2_.

### Statistical Analysis

Statistical analyses were performed using Prism statistical software (version 5.01; Graph-Pad Software, San Diego, CA). Nonparametric two-tailed Mann-whitney test was used to compare quantitative values. All *P* values were considered significant if less than 0.05. The data reported was pooled from three independent experiments, unless specified otherwise.
